# Public health round-up

**DOI:** 10.2471/BLT.15.011015

**Published:** 2015-10-01

**World falls short of child survival goal**

Child survival has improved with deaths of children halved from an estimated 12.7 million per year in 1990 to 5.9 million in 2015. The world has still fallen short of Millennium Development Goal four to reduce these deaths by two-thirds, according to the *Levels and trends in child mortality report 2015.* This photo shows children sleeping on mats while waiting to receive health services at Chagoua dispensary in N’Djamena, the capital of Chad. http://www.who.int/maternal_child_adolescent/documents/levels_trends_child_mortality_2015/en/

**Figure:**
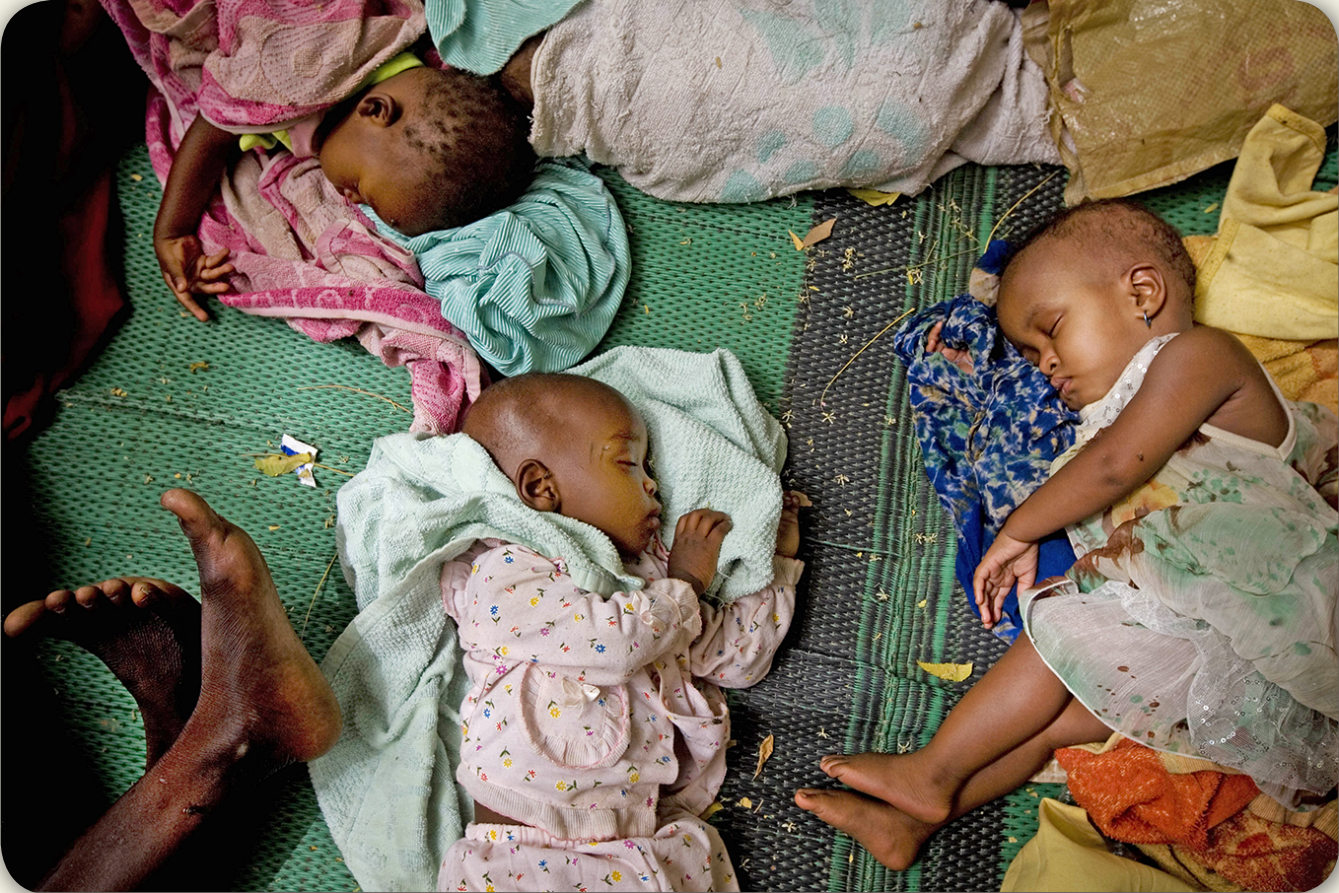
UNICEF/Kate Holt

## Call for action on nutrients

Vitamin and mineral deficiencies affect more than half of the world’s population but few countries have health programmes to address these by adding vitamins and minerals to staple foods.

Around 300 government officials, scientists, technical experts and donors from all over the world met last month in the Tanzanian city of Arusha to discuss how to use food fortification to prevent vitamin and mineral deficiencies leading to micronutrient malnutrition.

Micronutrient malnutrition impairs physical and mental development in children, reduces productivity in adults and has dire effects on pregnant and lactating women, and newborn babies.

However, vitamins and minerals can be added to staple foods such as food grade salt, wheat or maize flours, rice, vegetable cooking oils and some condiments to increase people’s intake of nutrients.

“There is a large body of scientific evidence showing that food fortification can help to correct micronutrient deficiencies,” said Dr Francesco Branca, WHO Director of Nutrition for Health and Development.

“Food fortification should be part of a comprehensive public health strategy to ensure adequate micronutrient supply to all the population, particularly vulnerable groups,” he said.

The meeting from 9 to 11 September was hosted by the Tanzanian Ministry of Health and nongovernmental organization GAIN.

http://www.gainhealth.org/events/future-fortified/

## WHO urges new approach to ageing and health

Profound changes are needed in the way health services are provided to meet the demands of rapidly ageing populations in many countries, according to the *World report on ageing and health*.

The report released by the World Health Organization (WHO) last month calls for the transformation of health systems from “disease-based curative models” into integrated care models that can respond to a wide range of older people’s needs.

Long-term care tends to be viewed as “a minimal and basic safety net” for older people who can no longer look after themselves. The report calls for a shift towards optimizing functional ability by building individual capacities and compensating for any lack of physical or mental function, for example, with mobility aids and other technology.

“The greatest costs to society are not the expenditures made to foster this functional ability, but the benefits that might be missed if we fail to make the appropriate adaptations and investments,” said Dr Margaret Chan, WHO Director-General, in the preface.

A change both in approach and attitude is required. The 246-page report launched on 30 September argues that common perceptions of the ageing process and what is needed to address older people’s needs are “stereotyped”, “outdated” and not backed by the latest scientific evidence.

Drawing on the evidence in the report, WHO proposes a global strategy and action plan on ageing and health for countries to better address the needs of older people. 

An online public consultation on the draft strategy is open until 30 October, (http://www.who.int/ageing/consultation/). The draft strategy will be discussed at a global consultation with Member States and other stakeholders on 29–30 October in Geneva, Switzerland.

http://www.who.int/ageing

## R&D for affordable treatment

Research and development (R&D) projects for hepatitis C, which affects 130 to 150 million people worldwide, and mycetoma, a chronic inflammation, will soon be launched, according to the new business plan of the Drugs for Neglected Diseases initiative (DNDi).

The partnership also said it would create an internal task force with WHO to look into establishing an R&D initiative to develop new antibiotics, given growing antibiotic resistance worldwide and the dearth of new candidate antibiotics in the drugs pipeline.

New effective drugs for hepatitis C are prohibitively expensive especially for people in low- and middle-income countries and an estimated 500 000 people die worldwide every year from hepatitis C-related liver diseases.

To develop affordable treatment for hepatitis C infection, DNDi said it would conduct clinical trials for drug combinations consisting of recently approved drugs in middle-income countries.

“The high cost of a new generation of drugs for hepatitis C has become one of the world’s most pressing and high-profile public health challenges, leaving millions of patients behind,” DNDi said.

For mycetoma, DNDi said it would test a promising drug candidate for this “devastating illness for which there has been virtually no R&D – leaving patients suffering from the effects of toxic and ineffective drugs”.

The independent not-for-profit partnership, which aims to develop affordable drugs, said it was launching new projects having transferred its malaria activities to another R&D partnership, Medicines for Malaria Venture.

With a pipeline of over 30 projects for some of the world’s most neglected diseases, DNDi aims to deliver 16 to 18 new treatments with an estimated total budget of €650 million (US$ 728 million) by its 20th anniversary in 2023.

The DNDi pipeline includes R&D projects for treatments for African trypanosomiasis, leishmaniasis, and Chagas disease as well as filarial diseases and paediatric HIV, according to its 2015–2023 business plan that was released last month.

http://www.dndi.org/

**Cover photo**

The vast influx of refugees and migrants to countries of the WHO European Region calls for an urgent response to their health needs. This month’s cover photo shows a young Syrian girl in the town of Gevgelija, the former Yugoslav Republic of Macedonia, waiting to board a train.

**Figure:**
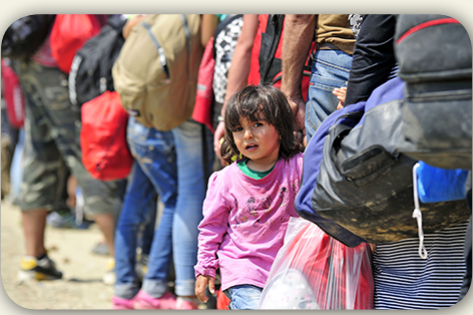
© UNICEF/Tomislav Georgiev

## Regulating traditional medicine

WHO has urged countries in Africa to regulate their traditional medicine practitioners to protect the public against potentially harmful practices.

Only 21 of the 47 countries in WHO’s African Region have laws or regulations governing traditional medicine practice, and even where such laws and regulations exist they are not always enforced.

An estimated 80% of people in African countries seek treatment from traditional health practitioners, according to WHO.

“The benefits of traditional medicine are evident to all,” said WHO Regional Director for Africa Dr Matshidiso Moeti, in her message on the 31 August, African Traditional Medicine Day.

“However, there is no doubt that proper regulation is essential to the provision of quality, safe and effective health-care products and services.”

“Strong regulatory systems are needed in countries to ensure that practitioners are properly trained and endorsed by a regulatory body,” Moeti said.

Such systems are particularly important in rural areas, where traditional medicine is sometimes the only kind of health care available.

WHO has developed a range of tools and guidelines that countries can use to regulate the sector including: a model legal framework for the practice of traditional medicine; a regulatory framework for traditional medicine practitioners, practices and products and a model code of ethics and practice for traditional health practitioners.

“I urge governments to protect the health of their citizens by prioritizing the establishment and strengthening of regulatory bodies for traditional health practitioners operating in the African Region,” Moeti said.

http://www.afro.who.int/en/rdo/speeches/4735-message-of-who-regional-director-for-africa-dr-matshidiso-moeti-on-african-traditional-medicine-day-2015.html

## BMA medical book awards

A WHO manual on best practices for safe abortion care won first prize in the obstetrics and gynaecology category of the British Medical Association medical books awards last month.

The manual entitled, *Clinical practice handbook for safe abortion*, was described by the jury as “concise, relevant and inviting to use with clear, helpful and accurate content”. The manual aims to guide practitioners in the application of clinical recommendations from the 2nd edition of *Safe abortion: technical and policy guidance of health systems*.

Another WHO book entitled, *Birth defects surveillance: a manual for programme managers*, produced in collaboration with the United States Centers for Disease Control and Prevention and the International Clearinghouse of Birth Defects Surveillance and Research was “highly commended” in the public health category of the medical book awards.

The jury praised the birth defects book for its “methodical approach first to principles behind a programme and then to categorization of defects” and its “clear unambiguous text is complemented by excellent use of colour figures and illustrations”. 

It is part of a three-document companion of which the other parts are an atlas and a facilitators’ guide.

http://www.who.int/nutrition/publications/birthdefects_manual/en/

**Looking ahead**

**11–13 October – World Health Summit of M8 Alliance of Academic Health Centers, Universities and National Academies. Berlin, Germany.** http://www.worldhealthsummit.org/

**29–30 October – Consultation on the global strategy and action plan on ageing and health, WHO HQ, Geneva, Switzerland.** http://www.who.int/ageing/consultation/en/

**18–19 November – Second Global High-Level Conference on Road Safety. Brasilia, Brazil.**

**30 November – 11 December – United Nations Climate Change Conference in Paris, France.** http://www.cop21.gouv.fr/en

